# Paeoniflorin Directly Targets ENO1 to Inhibit M1 Polarization of Microglia/Macrophages and Ameliorates EAE Disease

**DOI:** 10.3390/ijms26083677

**Published:** 2025-04-13

**Authors:** Ying Sun, Guojue Wang, Shengzhe Li, Yongshuai Jiang, Yunhui Liu, Yidan Gao, Yuanyang Yuan, Hong Nie

**Affiliations:** Shanghai Institute of Immunology, Department of Immunology and Microbiology, Shanghai Jiao Tong University School of Medicine, Shanghai 200025, China; sunyingying@sjtu.edu.cn (Y.S.); gjwang0422@sjtu.edu.cn (G.W.); shengzhe_li@sjtu.edu.cn (S.L.); jys_0225@163.com (Y.J.); linkinparkerny@163.com (Y.L.); gaoyidan_dana@sjtu.edu.cn (Y.G.)

**Keywords:** paeoniflorin, EAE, microglia/macrophage polarization, ENO1, glucose metabolism

## Abstract

The chronic autoimmune disease multiple sclerosis (MS) now remains incurable. Paeoniflorin (PF), which is a monoterpene glucoside obtained from *Paeonia lactiflora Pall*, is recognized for neuroprotective and anti-inflammatory properties. However, the precise mechanism by which PF regulates MS is unclear. This work aims to elucidate the underlying mechanisms of PF in EAE, a well established animal model of MS, and to discover the target proteins that PF directly acts on. Our results revealed that PF administration can significantly attenuate the clinical symptoms of EAE and alleviate the central nervous system (CNS) inflammatory environment by inhibiting M1-type microglia/macrophages. Mechanistically, PF was found to directly interact with the glycolytic enzyme α-enolase (ENO1), inhibiting its enzymatic activity and expression to impair glucose metabolism, thereby suppressing microglia/macrophage M1 polarization and ameliorating CNS inflammation. Significantly, *Eno1* knockdown in microglia/macrophages diminished their pro-inflammatory phenotype, while treatment with ENOBlock or the specific knockout of *Eno1* in microglia led to EAE remission, underscoring the critical role of ENO1 in EAE progression. This study uncovers the molecular mechanism of PF in treating EAE, linking the anti-inflammatory property of PF to the glucose metabolism process, which will broaden the prospective applications of PF.

## 1. Introduction

Multiple sclerosis (MS), a chronic inflammatory disease characterized by inflammation and demyelination of CNS, affects more than 2.3 million people globally [1]. Currently, MS is one of the most prevalent neurological disorders affecting young people, and several disease-modulatory therapies (DMTs) are approved to treat the expanding population [2]. However, the limited efficacy and financial burden of these DMTs underscore an urgent unmet medical need for discovering new therapeutic targets and improving the quality of life for MS patients [3,4].

MS has a complex multifactorial etiology involving disruption of the blood-brain barrier in CNS, infiltration of T cells and macrophages into the CNS, activation of resident microglia and astrocytes, along with other contributing factors [5]. Resident microglia cells and infiltrated macrophages in CNS have similar functions and biomarkers. Thus, they are collectively referred to as microglia/macrophages [6,7]. Autoantigen intake by dendritic cells or microglia/macrophages triggers cytokine and chemokine release, which in turn causes an influx of B cells and T cells. The response of T cells to antigen presentation and the particular cytokine microenvironment trigger Th1 and Th17 cell differentiation [8,9]. All these cells precipitate an inflammatory response that results in neurological damage [10]. Consequently, recent research focuses on targeting specific immune cells, such as Th1 cells, Th17 cells, and microglia/macrophages, for potential therapies for MS, aiming to address anti-neuroinflammation and restore the immune balance [11].

Activated immune cells undergo an alteration called metabolic reprogramming, which is characterized by a reduction in oxidative phosphorylation (OXPHOS) towards glycolysis, influencing their function and phenotype [12,13]. Glycolysis is essential for pro-inflammatory cells like M1 macrophages, while oxidative metabolism promotes the development of anti-inflammatory M2 macrophages and Treg cells [14,15]. The upregulation of glycolytic enzymes, pro-inflammatory cytokines (e.g., IL-1β), and glycolysis-related genes in MS patients suggests that enhanced glycolysis plays a significant role in MS pathogenesis. [16]. Previous studies have demonstrated that blocking pyruvate kinase M2 (PKM2), a crucial enzyme in glycolysis, could inhibit metabolic reprogramming through the mTOR pathway and decrease M1 polarization of macrophages [17]. The immunomodulatory drug Dimethyl fumarate (DMF) for MS and psoriasis treatment has recently been demonstrated to succinate and deactivate the catalytic cysteine of the glycolytic enzyme GAPDH. This action results in the downregulation of glycolysis in activated myeloid and lymphoid cells, thereby mediating anti-inflammatory effects [18]. Therefore, modulating the metabolism of immune cells emerges as a promising therapeutic strategy for the treatment of MS and other autoimmune diseases.

Paeoniflorin (PF), a bioactive compound extracted from Radix Paeoniae, demonstrates significant anti-inflammatory, antioxidant, neuroprotective, and metabolic regulatory effects in various murine models [19,20,21,22]. For instance, in the collagen-induced arthritis (CIA) mouse model, PF provides protection by suppressing pro-inflammatory cytokines, such as TNF-α, IL-6, and IL-17, and by restoring the balance between Th17 and Treg cells [23,24]. The ability of PF to penetrate the blood–brain barrier enhances its potential in treating CNS disorders [25]. In the context of MPTP-induced Parkinson’s disease, PF mitigates dopaminergic neurodegeneration by activating adenosine A1 receptors, resulting in a reduction in pro-inflammatory cytokines and the preservation of dopaminergic neurons [26]. Despite these advancements, most studies on the mechanisms of PF’s action have concentrated on cell phenotypes and the associated signaling pathways without identifying specific molecular targets.

Given its dual anti-inflammatory and neuroprotective properties, PF is proposed as a beneficial agent for MS. We employed the EAE model to assess the therapeutic potential of PF and to elucidate its regulatory effects on immune cells. A novel aspect of our research is the utilization of a comprehensive array of assays, including the non-labeled drug target protein screening technology DARTS, molecular docking, and gene knockdown and overexpression systems. These methodologies were employed to identify and validate the direct target and mechanism of action of PF for EAE treatment. Our systematic investigation revealed that PF significantly alleviates the clinical symptoms of EAE mice and modulates neuroinflammation by inhibiting the M1 polarization of microglia/macrophages. Following that, molecular docking combined with biological experiments demonstrated for the first time that ENO1 is the target of PF in regulating microglia/macrophage function. PF interacted with ENO1 and led to the downregulation of ENO1 enzymatic activity and expression, thus converting the activated microglia/macrophages to a steady state. ENOBlock treatment and the specific knockout of *Eno1* in microglia also effectively reduced the clinical score of EAE mice. These findings collectively suggest that PF is a promising drug candidate for MS, and the efforts on ENO1 validation might also be helpful to provide an effective drug target for future therapy.

## 2. Results

### 2.1. PF Ameliorates Clinical Symptoms in EAE Mice

**Figure 1:**
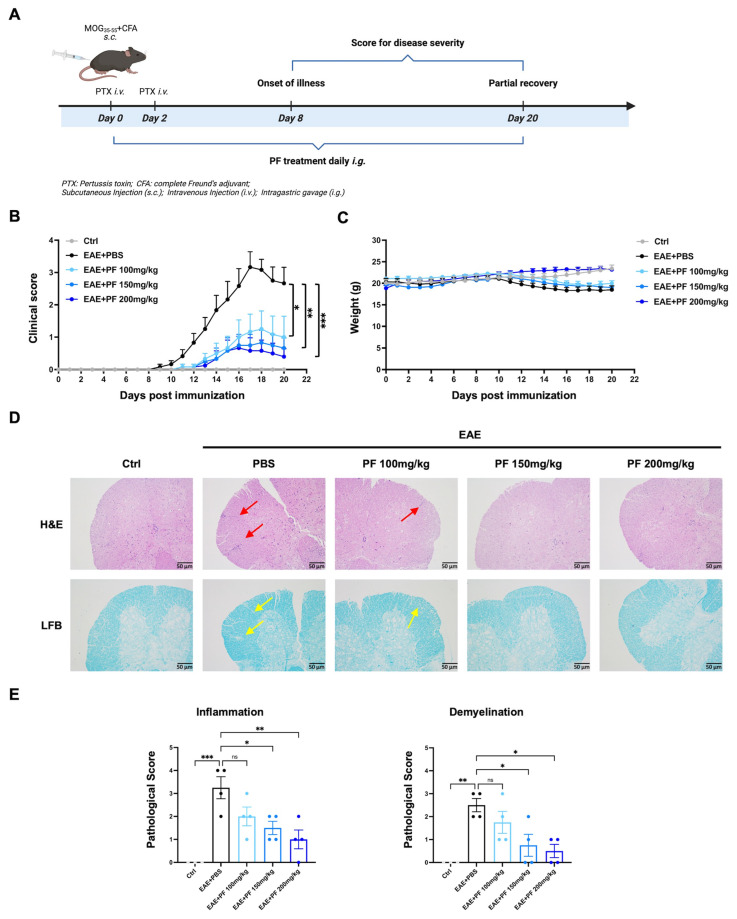
PF administration alleviates clinical symptoms, CNS inflammation, and demyelination in EAE mice. (**A**) Schematic diagram of MOG-induced EAE and PF administration. (**B**,**C**) Clinical scores (**B**) and body weights (**C**) of wild-type control mice (*n* = 3), vehicle PBS-treated EAE mice and PF-treated EAE mice (100 mg/kg, 150 mg/kg, 200 mg/kg) (*n* = 6), PF and PBS treatment initiated from day 0 post immunization. (**D**) Representative H&E staining and LFB staining of lumbar spinal cords obtained from wild-type control mice (*n* = 3) and EAE mice treated with PBS or PF on day 16 post immunization (*n* = 4) (scale bar: 50 μm), red arrows indicate immune cell infiltration and yellow arrows indicate demyelination. (**E**) Quantitative pathology scores for inflammation (H&E) and demyelination (LFB). Data are presented as mean ± SEM. * *p* < 0.05, ** *p* < 0.01, *** *p <* 0.001; ns, no significance.

To assess the therapeutic efficacy of PF in EAE mice, we generated the MOG-induced EAE model, followed by intragastric administration with PF as treatment or PBS as vehicle control from the day of immunization (Figure 1A). We observed that PF significantly reduced the EAE clinical severity in a dose-dependent manner compared to the vehicle treatment (Figure 1B) with no apparent effect on the mice’s body weights (Figure 1C). We examined the impact of PF on inflammation and demyelination in the lumbar spinal cord of EAE mice at the peak stage (day 16 post immunization) using H&E and LFB staining. The results showed that the PBS-treated EAE mice exhibited inflammatory cell infiltration accompanied by demyelination, and these damages were significantly ameliorated by PF treatment (150 mg/kg and 200 mg/kg) (Figure 1D,E). These findings suggest that PF treatment attenuates the disease symptoms of MOG-induced EAE mice.

### 2.2. PF Inhibits the Polarization of Microglia/Macrophages to M1 Phenotype

**Figure 2:**
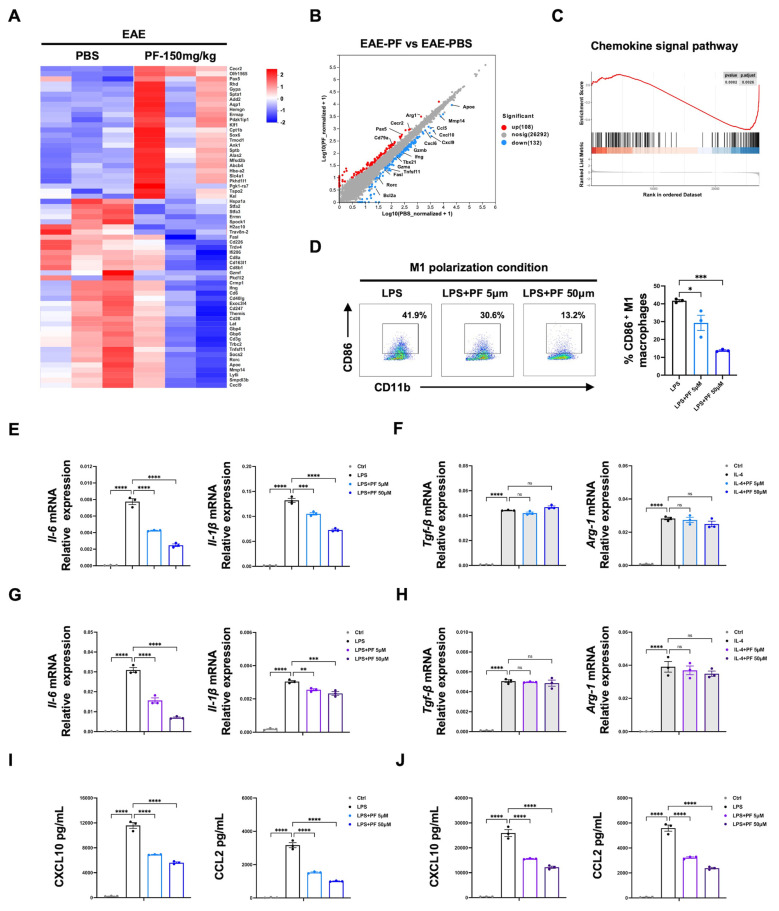
PF inhibits the M1-type polarization of microglia/macrophages in vitro. (**A**–**C**) The CNS mononuclear cells were isolated from EAE mice treated with PBS or PF (150 mg/kg) on day 16 post immunization (*n* = 3). (**A**) Heatmap of the representative up- and downregulated mRNA of CNS mononuclear cells. (**B**) Volcano diagram of the top upregulated and downregulated genes and (**C**) representative GSEA plot (compared to EAE-PBS group) from RNA-seq analysis. (**D**–**H**) BMDMs and BV2 cells were treated with PF in M1 polarization condition (LPS 100 ng/mL) or M2 polarization condition (IL-4 20 ng/mL) for 24 h. The percentages of CD86^+^ M1 BMDMs (**D**) were analyzed by flow cytometry (*n* = 3). The mRNA levels of *Il-6* and *Il-1β* in M1 BMDMs (**E**) or M1 BV2 cells (**G**), and *Arg-1* and *Tgf-β* in M2 BMDMs (F) or M2 BV2 cells (H) were analyzed by qPCR (*n* = 3). (**I**,**J**) CXCL10 and CCL2 secretion in supernatants of M1-polarized BMDMs (**I**) and BV2 cells (**J**) with or without PF treatment for 24 h, measured by ELISA (*n* = 3). Data are expressed as the mean ± SEM. * *p* < 0.05, ** *p* < 0.01, *** *p* < 0.001, **** *p* < 0.0001; ns, no significance.

To discover the mechanism of PF on EAE, CNS-infiltrating mononuclear cells from PBS- or PF-treated EAE mice (150 mg/kg) were isolated, and RNA-seq analyses were conducted. There was a decrease in genes associated with T cell differentiation and chemotaxis, including *Tbx21*, *Mmp14*, *Ccl5*, *Cxcl9*, and *Cxcl10*, while there was an increase in macrophage-related genes such as *Arg-1* and *Cecr2* in the PF treatment group (Figure 2A,B). The GSEA results also exhibited significant changes in the chemokine-related pathway (Figure 2C). We then studied how PF regulates T cell or macrophage function. In vitro experiments showed that different concentrations of PF did not significantly impact the proliferation and apoptosis of CD4^+^ T cells (Figure S1A,B). We also examined the effect of PF on the differentiation of CD4^+^ T cells. PF showed no direct impact on the in vitro differentiation of Th1, Th17, or Treg cells (Figure S1C).

Next, we explored whether PF could regulate microglia/macrophage polarization in vitro using BMDMs and BV2 cell line as models. Our results showed that PF significantly inhibited CD86 expression on M1 polarized BMDMs (Figure 2D) and maintained consistent levels of CD206 on M2 polarized BMDMs (Figure S2). PF significantly reduced the mRNA expression of M1-associated cytokines *Il-6* and *Il-1β* in a dose-dependent manner (Figure 2E), while it did not alter the expression levels of M2-related genes, such as *Arg-1* and *Tgf-β* (Figure 2F). PF also showed a similar effect on BV2 cells (Figure 2G,H). Another hallmark of M1 polarized microglia/macrophages is the production of chemokines. The results also showed significantly reduced protein secretion of M1-related chemokines CCL2 and CXCL10 in BMDMs (Figure 2I) and BV2 cells (Figure 2J). All these findings indicate that PF suppresses M1 polarization of microglia/macrophages, thus inhibiting the inflammation process.

### 2.3. PF Alleviates CNS Inflammation in EAE Mice by Reducing the Percentages of M1 Microglia/Macrophages and Pathogenic T Cells

**Figure 3:**
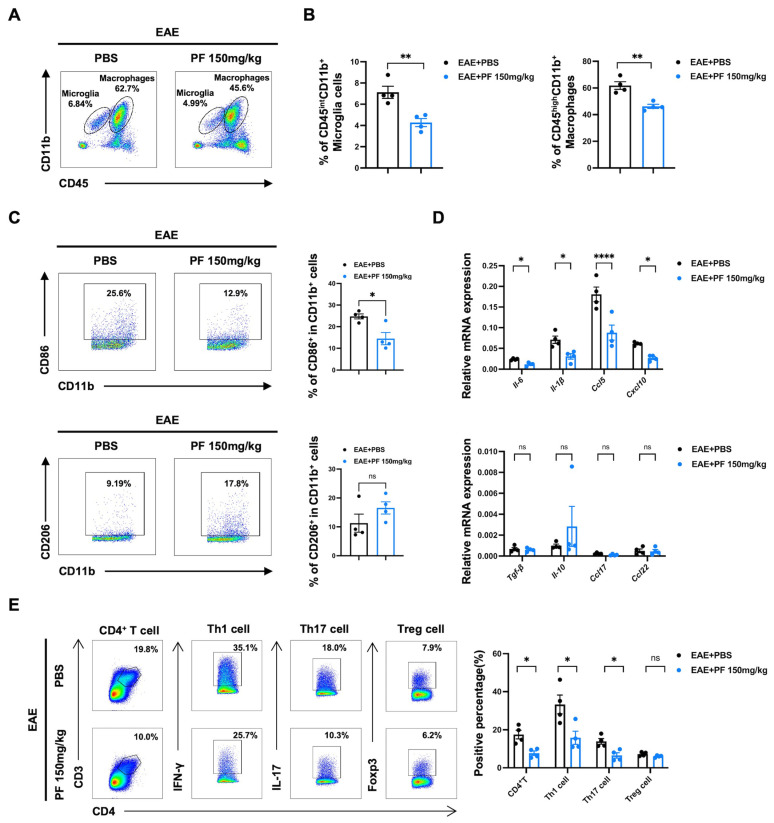
PF reduces the percentages of M1 microglia/macrophages and pathogenic T cells in the CNS of EAE mice. The CNS mononuclear cells were isolated from the EAE-PBS and EAE-PF group mice on day 16 post immunization. (**A**,**B**) Flow cytometry analysis of CD45^int^ CD11b^+^ microglia cells and CD45^high^ CD11b^+^ macrophages in CNS from the two group mice (*n* = 4). (**C**) Flow cytometry analysis of CD86^+^ or CD206^+^ cells in CD11b^+^ cells from the two group mice (*n* = 4). (**D**) The mRNA expression levels of cytokines and chemokines in CNS mononuclear cells from the two group mice were determined by qPCR (*n* = 4). (**E**) The percentage of CD4^+^ T cells and T-helper cells such as Th1, Th17, and Treg cells in the CNS were analyzed by flow cytometry (*n* = 4). Data are expressed as the mean ± SEM. * *p* < 0.05, ** *p* < 0.01, **** *p* < 0.0001; ns, no significance.

For the in vivo experimentation revealed in Figure 3A,B, PF treatment reduced the percentages of CD11b^+^ cells in the CNS, including microglia (CD45^int^ CD11b^+^ cells) and macrophages (CD45^high^ CD11b^+^ cells). Additionally, two populations of CD11b^+^ cells were further examined. The CD11b^+^ CD86^+^ M1 subset exhibited a decreased percentage following PF treatment, whereas the CD11b^+^ CD206^+^ M2 proportion remained relatively stable (Figure 3C). Real-time PCR was used to evaluate the mRNA expression levels of markers associated with the M1 phenotype (*Il-6*, *Il-1β*, *Ccl5*, *Cxcl10*) and M2-phenotype genes (*Tgf-β*, *Il-10*, *Ccl17*, *Cxcl22*) in the CNS mononuclear cells. PF administration downregulated M1 phenotype markers with no significant change in M2-phenotype genes in EAE mice (Figure 3D). In the CNS of EAE mice, PF therapy reduced the expression of *Ccl5* and *Cxcl10*, suggesting that PF may lessen peripheral T cell migration into the CNS. We discovered that PF treatment decreased the proportions of CD4^+^ T cells, including Th1 and Th17 cells, in the CNS of EAE mice (Figure 3E). These findings suggest that PF reduces CNS inflammation by decreasing the degree of M1 polarization of CNS microglia/macrophages and lowering the proportion of pathogenic T cells within EAE mice.

### 2.4. PF Targets ENO1 and Reduces Enzyme Activity as Well as Glycolysis Status of M1 Microglia/Macrophages

**Figure 4:**
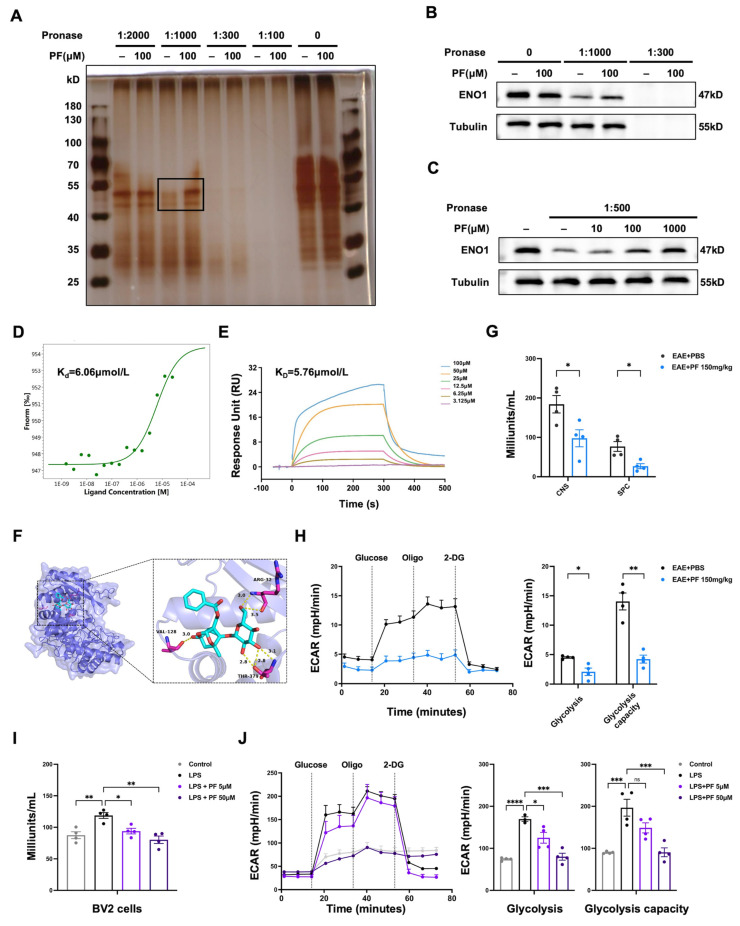
PF targets ENO1 and reduces enzyme activity as well as glycolysis status. (**A**) DARTS assay and silver staining were performed to detect changed protein bands upon PF incubation in Pronase digested BV2 cell lysates. The changed protein bands (black-boxed) were processed for mass spectrometry. (**B**,**C**) PF-ENO1 binding was validated by DARTS and WB. (**D**) Binding of PF to ENO1 was determined with MST assay. (**E**) SPR assay tested the binding affinity of PF to ENO1. (**F**) Molecular docking results of ENO1 with PF. The model image was generated by PyMOL version 2.5.0 (Schrödinger, LLC, New York, NY, USA). ENO1 was shown as a slate cartoon, PF as a cyan stick, and binding site residues as magenta sticks. Hydrogen bonds were depicted with yellow dashed lines. (**G**) CD11b^+^ cells were isolated from CNS mononuclear cells or spleen cells in PBS-EAE or PF-EAE mice on day 16 after immunization. Enolase enzyme activity was monitored by the Enolase Activity Assay Kit (*n* = 4). (**H**) CD11b^+^ cells were isolated from CNS mononuclear cells in PBS-EAE or PF-EAE mice on day 16 after immunization. Glycolysis status of isolated CD11b^+^ cells were monitored by ECAR assay. (**I**,**J**) The enolase enzyme activity of BV2 cells was analyzed by the Enolase Activity Assay Kit (**I**) and the glycolysis status were monitored by ECAR assay (**J**) (*n* = 4). Data are presented as the mean ± SEM. * *p* < 0.05, ** *p* < 0.01, *** *p* < 0.001. **** *p* < 0.0001; ns, no significance.

According to our in vitro and in vivo research findings, PF can attenuate CNS inflammation through the direct inhibition of M1 polarization in microglia/macrophages. Furthermore, elucidating and confirming the biological mechanism of a drug requires establishing a link between its effects and its specific target. So, we aimed to determine the molecular target of PF in microglia/macrophages by conducting Drug Affinity Responsive Target Stability (DARTS) assays. The findings revealed ENO1 as a highly enriched protein in the PF-treated sample (Figure 4A, Table S1). The specific interaction between PF and ENO1 was further confirmed through DARTS and Western blot (Figure 4B,C). Additional assays, including MST and SPR, further validated the binding equilibrium Kd of 6.06 μM (Figure 4D) and binding affinity (K_D_) of 5.76 μM (Figure 4E) between PF and ENO1. To predict the potential binding site of PF on ENO1, a series of molecular dynamics simulations were performed using the ENO1 structure (PDB ID: 3B97) in complex with PF. Molecular docking analysis indicated that PF may form a covalent interaction with ENO1, exemplified by a hydrogen bond involving VAL128. With these interaction forces, the binding energy of PF-ENO1 complex was −7.5 kcal/mol (Figure 4F). These findings suggest that PF could impact the transition between open and closed conformations of ENO1, thereby modulating its activity.

Since ENO1 is a glycolytic enzyme, we explored whether the binding of PF to the protein affects the enzymatic activity. As shown in Figure 4G, CD11b^+^ cells isolated from CNS or spleen of PF-treated EAE mice showed reduced ENO1 enzyme activity compared to PBS-treated EAE mice. Simultaneously, the measurement of the extracellular acidification rate (ECAR) demonstrated that PF treatment significantly decreased both ECAR and glycolytic flux in CD11b^+^ cells isolated from the CNS mononuclear cells of EAE mice (Figure 4H). Furthermore, PF inhibited the enzymatic activity of ENO1 (Figure 4I) and modulated ECAR levels (Figure 4J) in a dose-dependent manner in BV2 cells. These findings suggest that ENO1 may serve as a potential anti-inflammatory target of PF.

### 2.5. Downregulation of ENO1 Contributes to Anti-Inflammatory Function of PF in M1 Microglia/Macrophages

**Figure 5:**
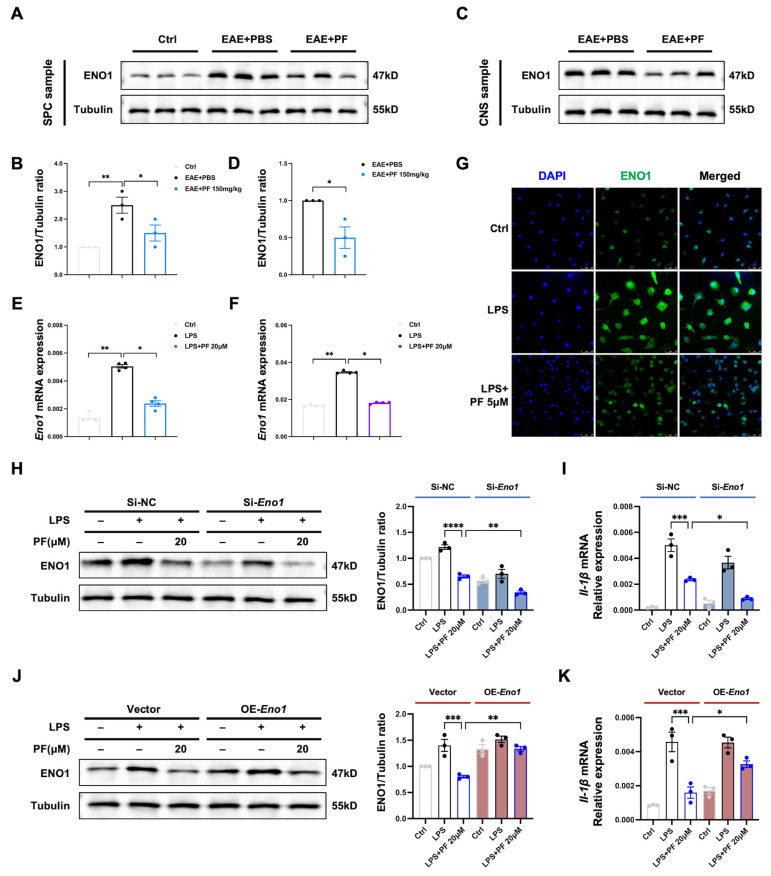
PF inhibits ENO1 expression in M1 microglia/macrophages accompanied by decreased IL-1β expression. (**A**,**B**) CD11b^+^ cells were isolated from spleen cells of wild-type mice, EAE-PBS and EAE-PF (150 mg/kg) group mice. ENO1 protein expression levels were analyzed through western blot (*n* = 3). (**C**,**D**) CD11b^+^ cells were isolated from CNS mononuclear cells in EAE-PBS and EAE-PF (150 mg/kg) group mice on day 16 post immunization. The expression levels of ENO1 protein were detected by western blot (*n* = 3). (**E**,**F**) The mRNA expression of *Eno1* in LPS-stimulated BMDMs (**E**) and BV2 cells (**F**) after PF treatment were analyzed by qPCR (*n* = 4). (**G**) Protein localization of ENO1 in LPS-stimulated BMDMs were detected by immunofluorescence (scale bar: 25 μm). (**H**–**K**) RAW 264.7 cells were transfected with si-NC or si-*Eno1* and control plasmid or *Eno1* overexpression (OE) plasmid for 48 h, then the cells were collected after stimulation by LPS and PF treatment for 24 h. ENO1 protein expression levels were analyzed through western blot (**H**,**J**), and the mRNA expression levels of *IL-1β* were analyzed by qPCR (**I**,**K**). Data are presented as the mean ± SEM. * *p* < 0.05, ** *p* < 0.01, *** *p* < 0.001, **** *p* < 0.0001.

Previous research has demonstrated that microglia cells upregulate the expression of glycolytic enzymes, such as hexokinase 2 (HK2) and phosphoglycerate kinase (PGK1), in response to pro-inflammatory stimuli, while there lacks clear evidence of ENO1 [27,28]. In this study, we took a look at changes in ENO1 expression levels during EAE progression and microglia cell activation. We found that ENO1 levels in peripheral macrophages isolated from the spleen cells of EAE mice were much higher than those from wild-type mice, and ENO1 expression decreased from PF-treated EAE group compared with the PBS-treated EAE group (Figure 5A,B). ENO1 abundance in CNS isolated microglia/macrophages from PF-treated EAE group showed the same decreased trend when compared to PBS-treated EAE group (Figure 5C,D). Then, the in vitro experiments also showed increased *Eno1* gene expression during M1 polarization of BMDMs (Figure 5E) and BV2 cells (Figure 5F), and PF treatment leading to decreased *Eno1* mRNA level (Figure 5E,F) and protein level (Figure 5G).

Next, *Eno1* was knocked down or overexpressed in macrophage cell line RAW 264.7 to further validate the function of ENO1, a key enzyme of glycolysis, in microglia/macrophage polarization following PF treatment. The results showed that decreased ENO1 level by PF treatment or transfection with *Eno1* siRNA led to reduced ENO1 protein expression (Figure 5H) and *Il-1β* mRNA expression (Figure 5I). Moreover, the sustained application of PF combined with *Eno1* siRNA resulted in a further reduction in ENO1 protein expression and *Il-1β* mRNA expression (Figure 5H,I). Conversely, RAW 264.7 cells were transfected with either an *Eno1* overexpression plasmid or an empty vector, followed by incubation with PF in the presence of LPS. Notably, *Eno1* overexpression counteracted the inhibitory effects of PF on ENO1 protein expression (Figure 5J) and *Il-1β* mRNA expression (Figure 5K). These results indicate that the downregulation of ENO1 plays a key role in the anti-inflammatory action of PF in M1 microglia/macrophages.

### 2.6. ENOBlock Treatment or Specific Knockout of Eno1 in Microglia Inhibits M1 Polarization and Alleviates EAE Progression

**Figure 6:**
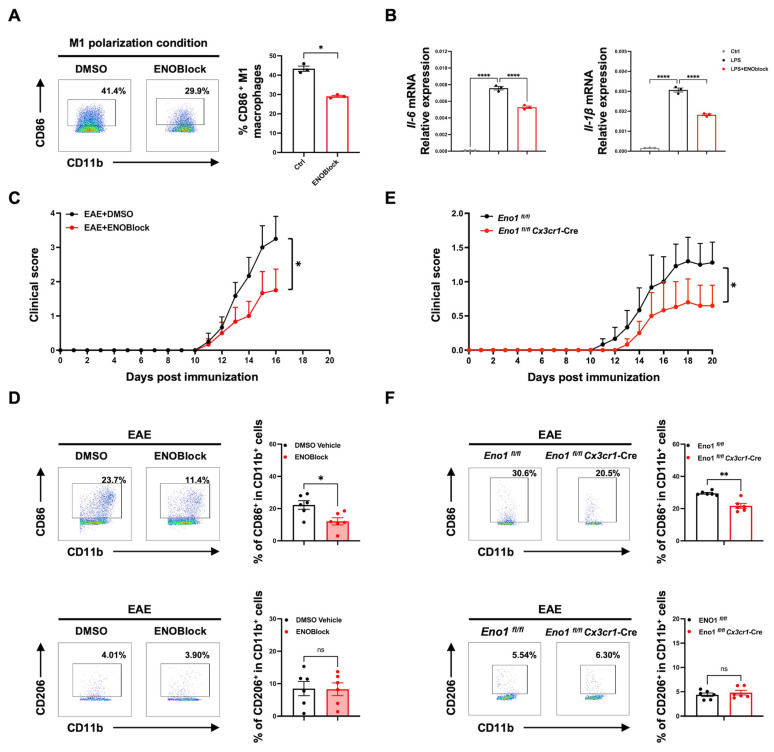
ENOBlock treatment or specific knockout of *Eno1* in microglia inhibits M1 polarization and alleviates EAE progression. (**A**,**B**) BMDMs were treated with ENOBlock (20 μM) in M1 polarization condition (LPS 100 ng/mL) for 24 h (*n* = 3). The percentage of CD86^+^ M1 BMDMs was analyzed by flow cytometry (**A**), and the mRNA expression of *Il-6* and *Il-1β* in M1 BMDMs was analyzed by qPCR (**B**). (**C**) The clinical score of MOG-induced EAE mice treated with DMSO vehicle or ENOBlock (5 mg/kg) intraperitoneally from day 5 post immunization (*n* = 6). (**D**) Flow cytometry analysis of CNS mononuclear cells isolated from DMSO vehicle and ENOBlock-treated mice on day 16 post immunization. Representative data and percentages of CD86^+^ or CD206^+^ cells in CD11b^+^ cells in CNS from the two group mice (*n* = 6). (**E**) The clinical score of MOG-induced *Eno1^fl/f^^l^* and *Eno1 ^fl/f^^l^ Cx3cr1-Cre* EAE mice (*n* = 6). (**F**) CNS mononuclear cells isolated from *Eno1^fl/f^^l^* and *Eno1^fl/f^^l^ Cx3cr1-Cre* EAE group mice on day 16 post immunization were analyzed by flow cytometry. Representative data and percentages of CD86^+^ or CD206^+^ cells in CNS from the two group mice (*n* = 6). Data are presented as the mean ± SEM. * *p* < 0.05, ** *p* < 0.01, **** *p* < 0.0001; ns, no significance.

In order to clarify the involvement of ENO1 in M1 polarization of microglia/macrophages and inflammatory response in EAE, BMDMs were treated with ENOBlock in the presence of LPS. The results showed that ENOBlock significantly attenuated the percentage of CD86^+^ (M1 phenotype) cells and mRNA level of M1-type cytokines *Il-6* and *Il-1β* in BMDMs (Figure 6A,B). The in vivo administration of ENOBlock (5 mg/kg) alleviated the clinical severity of EAE (Figure 6C). This treatment also resulted in a decreased percentage of CD86^+^ CD11b^+^ M1 microglia/macrophages compared to the DMSO vehicle group (Figure 6D). However, no significant change was observed in the percentage of CD206^+^ M2 microglia/macrophages (Figure 6D).

To generate microglia-specific *Eno1* conditional knockout mice, we crossed *Eno1^fl/fl^* mice with *Cx3cr1-*Cre mice and subsequently induced the EAE model. The results revealed that the targeted deletion of *Eno1* in microglia could lead to a significant decrease in disease severity and delayed onset of EAE (Figure 6E). An analysis of the immune cell subpopulations revealed a decrease in CD86^+^ M1 microglia/macrophages in the CNS, while the levels of CD206^+^ M2 microglia/macrophages remained unchanged (Figure 6F). Thus, targeting ENO1 can alleviate M1 polarization of microglia/macrophages, thereby alleviating the inflammatory response associated with EAE.

## 3. Discussion

MS is a disease that affects young and middle-aged individuals, causing inflammation and disability. Currently, most approved drugs for MS are DMTs, with limited therapeutic efficacy and high cost [2]. Consequently, developing efficient therapeutic medicines is crucial to advance MS management. In recent years, there has been an increasing focus on exploring the therapeutic properties of natural ingredients for various diseases treatment. Previous studies have demonstrated that PF has marked anti-inflammatory, antioxidant, and neuroprotective effects [20,21,22]. However, the target proteins that PF directly acts on are unclear. Therefore, this study aimed to assess how effective PF is in treating EAE and identify the specific target which PF binds.

The etiology of MS is complex, with prevailing theories indicating that the disease is predominantly impacted by a variety of immune cells, such as self-reactive T and B cells, as well as activated microglia/macrophages, resulting in disease initiation and progression [11]. In this study, in vivo investigations utilizing flow cytometry on mononuclear cells derived from the CNS revealed a significant reduction in the populations of CD86^+^ M1 microglia/macrophages, as well as Th1 and Th17 cell subsets, following PF administration. In vitro analyses demonstrated that PF markedly inhibited M1 polarization and the expression of pro-inflammatory chemokines/cytokines in BV2 cells and BMDMs, while exerting no noticeable effect on T cell differentiation and proliferation. In addition, PF treatment reduced the expression of chemokine *Ccl5* and *Cxcl10* in the CNS of EAE; this finding suggests that the modulation of these chemokines may contribute to the observed reduction in pathogenic T cell infiltration within the CNS. Through the above research, we elucidated that PF could mitigate CNS inflammation by directly inhibiting M1 polarization in microglia/macrophages and indirectly reducing T cell infiltration; these combined effects contribute to an enhanced resistance to EAE disease.

A key finding in our research on PF’s regulatory mechanism is identifying its direct targets. Using mass spectrometry and DARTS, we initially identified ENO1 as one of the potential interacting proteins of PF. This was subsequently confirmed through MST, SPR, and Western blot. Molecular docking further suggested ENO1 as a crucial target in response to PF treatment. Although ENO1 has been extensively investigated in cancer biology [29], its involvement in CNS inflammation such as EAE and MS has not been elucidated. Our data proved that ENO1 plays a crucial role in CNS inflammation as evidenced by increased ENO1 enzyme activity, enhanced glycolytic status, and the upregulation of mRNA and protein level. Genetic approaches showed that reducing ENO1 via siRNA decreased *Il-6* and *Il-1β* mRNA expression in macrophages and conditional *Eno1* deletion in microglia significantly attenuated EAE severity. Other interventions such as ENOBlock administration inhibited M1 polarization, reduced cytokine *Il-6* and *Il-1β* gene expression, and mitigated EAE progression. These findings highlight ENO1 as a key metabolic–immune regulator in microglia/macrophage activation and EAE progression.

By revealing the critical role of ENO1, we further verified that PF alleviates EAE through the interaction and regulation of ENO1. PF administration significantly reduced ENO1 enzyme activity, leading to decreased glycolytic activity both in vitro and in vivo. This was accompanied by a notable downregulation of ENO1 expression during M1 polarization of microglia/macrophages and EAE progression. The inhibitory effect of PF on ENO1 expression can be enhanced by siRNA or reversed by *Eno1* overexpression, confirming PF’s regulation on microglial activation and EAE through ENO1 interaction. Collectively, PF emerges as a promising therapeutic agent for EAE.

Despite these advances, several questions still warrant further investigation. ENO1 transmits glycolytic signals to induce the production of inflammatory cytokines. However, the signaling cascade that connects the ENO1-mediated glycolytic pathway to immune activation signaling has yet to be fully elucidated. Furthermore, the PF–ENO1 interaction may not be specific for microglia/macrophage activation or limited to EAE and MS, as it could represent a general characteristic of inflammatory conditions. Further studies are necessary to elucidate the specific role of ENO1 in various immune cells and other inflammatory diseases.

Overall, our work highlights ENO1 as a new therapeutic target and key link between metabolism and immunity in inflammation. The natural compound PF ameliorates EAE disease and microglia/macrophage activation via specifically inhibiting this glycolytic enzyme activity.

## 4. Materials and Methods

### 4.1. Experimental Animals

C57BL/6 wild-type mice, aged between 6 and 8 weeks, were purchased from Lingchang Biotech (Shanghai, China). Specific *Eno1* knockout (*Eno1^fl/fl^ Cx3cr1*-Cre) mice were generated by crossing *Eno1^fl/fl^* mice with *Cx3cr1*-Cre mice, which were purchased from Cyagen (Suzhou, China). All mice were kept in a pathogen-free environment at Shanghai Jiao Tong University School of Medicine, and the Institutional Animal Care and Use Committee (IACUC) approved all experiment procedures (A-2022-013).

### 4.2. EAE Induction, PF Treatment, and ENOBlock Administration

To induce EAE, male C57BL/6 mice aged 6–8 weeks were subcutaneously injected with MOG_35–55_ peptide (GL Biochem (Shanghai) Ltd., Shanghai, China) mixed in CFA. Pertussis toxin (PTX), at a dose of 200 ng from List Labs (Campbell, CA, USA) or Merck (Darmstadt, Germany), was injected intravenously on day 0 and day 2 post immunization.

Paeoniflorin (PF, C_23_H_28_O_11_, with a purity of 98%) was procured from Yilin Biological Technology (Shanghai, China). For PF treatment, PF (100 mg/kg, 150 mg/kg, and 200 mg/kg) or control (PBS) were administered intragastrically (i.g.) at a volume of 200 μL daily from day 0 post immunization. For the administration of ENOBlock (MCE, Monmouth Junction, NJ, USA), EAE mice were assigned to two groups and received intraperitoneal injections of either ENOBlock (5 mg/kg, stocked in DMSO and further diluted in PBS) or DMSO (diluted in PBS) starting from day 5 post immunization. Mice were weighed daily and assessed for disease severity as previously described [30].

### 4.3. Histopathological Analysis of Spinal Cords

The lumbar spinal cord samples were fixed with 4% paraformaldehyde (PFA) and sliced at 5 μm thickness after being embedded in paraffin. These samples were stained with H&E to evaluate inflammatory cell infiltration and with LFB to assess demyelination. The pathological severity of the tissue was assessed and divided into five grades using Image Pro software 6.0, provided by Servicebio (Wuhan, China).

### 4.4. Cell Isolation

Mononuclear cells infiltrating the CNS were extracted from spinal cord and brain tissues utilizing Percoll (GE Healthcare, Chicago, IL, USA) gradient centrifugation. The mononuclear cells were collected from the interface between 37 and 70% Percoll. Cell suspensions from spleen were obtained and the red blood cells were then removed by ACK lysis. CD11b^+^ cells were subsequently isolated from these suspensions employing EasySep™ Mouse CD11b Positive Selection Kit (STEMCELL Technologies Inc., Vancouver, BC, Canada), while CD4^+^ T cells were isolated using EasySep™ Mouse CD4^+^ T Cell Isolation Kit (STEMCELL Technologies Inc., Vancouver, BC, Canada).

### 4.5. EAE CNS Mononuclear Cell RNA Sequencing and Analysis

RNA samples were isolated from CNS-infiltrating mononuclear cells of EAE animals treated with PBS or PF by TRIzol (Invitrogen, Carlsbad, CA, USA). RNA sequencing was conducted by Majorbio (Shanghai, China) using Illumina HiSeq 6000 platform (Illumina, San Diego, CA, USA). Adjusted *p*-value < 0.05 and |log2FC| ≥ 1 of the genes were identified by DESeq to be significantly differentially expressed. Expression profiles of different mRNAs after PF treatment were depicted by heatmaps, volcano plots, and GSEA.

### 4.6. Flow Cytometry Analysis

Fluorescent antibodies against surface markers (Thermo Fisher Scientific, Waltham, MA, USA; BD Biosciences, San Jose, CA, USA) were used to stain the cells in FACS buffer. For intracellular staining, the cells were triggered using a cell stimulation cocktail (Thermo Fisher Scientific, Waltham, MA, USA) and fixed and permeabilized using the Foxp3 Staining Buffer Set (Thermo Fisher Scientific, Waltham, MA, USA) or BD Cytofix/Cytoperm (BD Biosciences, San Jose, CA, USA), and then stained with fluorescent antibodies. Following washing, the cells were transferred to the BD LSRFortessa X20 (BD Biosciences, San Jose, CA, USA), and FlowJo software Version 10 (BD Biosciences, San Jose, CA, USA) was used to analyze the data. Detailed antibody information is listed in Table S2.

### 4.7. CD4^+^ T Cell Proliferation, Apoptosis, and Differentiation Assay

A total of 2 × 10^5^ CD4^+^ T cells were cultured in RPMI 1640 (Thermo Fisher Scientific, Waltham, MA, USA) complete medium. The cells were stimulated with 2 μg/mL plate-bound anti-CD3e monoclonal antibody (mAb) and 1 μg/mL anti-CD28 mAb (BD Biosciences, San Jose, CA, USA) for 48 h in the presence of PF treatment. T cell proliferation was evaluated using FITC-Ki67 antibody while T cell apoptosis analysis was performed with an annexin V/Dead Cell Apoptosis Kit (Thermo Fisher Scientific, Waltham, MA, USA). For T cell differentiation, 2 × 10^5^ CD4^+^ T cells were cultured with corresponding medium for 72 h: Th1 differentiation medium was supplemented with the same concentration of anti-CD3e/CD28 mAbs, anti-IL-4 (10 µg/mL), IL-12 (10 ng/mL), as well as IL-2 (10 ng/mL); Th17 differentiation media were mixed according to the CellXVivo Th17 Cell Differentiation Kit (R&D Systems, Inc., Minneapolis, MN, USA); for Treg cell differentiation, the same concentration of anti-CD3e/CD28 mAbs, anti-IL-4 (10 µg/mL), and anti-IFN-γ (10 µg/mL), TGF-β (15 ng/mL), IL-2 (10 ng/mL) were included. The cytokines and antibodies used were purchased from Peprotech (Cranbury, NJ, USA), BD Biosciences (San Jose, CA, USA), and Thermo Fisher Scientific (Waltham, MA, USA). PF (0 µM, 5 µM, 50 µM) was added to the mixed media and the cells were collected and detected by flow cytometry.

### 4.8. Microglia/Macrophage Polarization Assay

Bone marrow-derived macrophages (BMDMs) were obtained according to a previously established protocol and cultured in α-MEM medium (Thermo Fisher Scientific, Waltham, MA, USA) with 10% FBS supplemented with 20 ng/mL of M-CSF (Peprotech, Cranbury, NJ, USA). On day 6, the cells were stimulated with 100 ng/mL LPS (Sigma-Aldrich, St. Louis, MO, USA) for M1 polarization or 20 ng/mL IL-4 (Peprotech, Cranbury, NJ, USA) for M2 polarization for 24 h. Subsequently, BMDMs were collected for further analysis. Mouse BV2 microglia cell line and RAW 264.7 macrophage cell line were obtained from the Cell Bank of Type Culture Collection of Chinese Academy of Sciences. The cells were cultured in DMEM medium (Thermo Fisher Scientific, Waltham, MA, USA) with 10% FBS. Then, 5 × 10^5^ cells were seeded in 6-well plates for 24 h, and the cells were treated with PF in M1 or M2 polarization condition for 24 h. Subsequently, BV2 cells or RAW 264.7 cells were collected for further analysis.

### 4.9. Immunofluorescence

BMDMs were cultured on coverslips in 6-well plates with LPS and PF treatment. After fixation with paraformaldehyde, the cell membranes were permeabilized using Triton-X-100. Sections were blocked using 5% BSA and incubated with anti-ENO1 antibodies (Abcam, Cambridge, UK) at 4 °C overnight followed by the application of Alexa Fluor 488 labeled secondary antibody (Abcam, Cambridge, UK) and DAPI (Thermo Fisher Scientific, Waltham, MA, USA). The localization of ENO1 was examined using a confocal microscope.

### 4.10. Quantitative PCR (qPCR)

Total RNA from BMDMs, BV2 cells, and isolated mononuclear cells were extracted using TRIzol, then cDNA was synthesized with PrimeScript RT Master Mix (TaKaRa Bio Inc., Kusatsu, Shiga, Japan) according to the manufacturer’s procedure. Quantitative PCR was executed using SYBR Green Master Mix (Yeasen Biotechnology (Shanghai) Co., Ltd., Shanghai, China), then PCR amplification was conducted using ViiA7 Real-Time PCR System (Applied Biosystems (Thermo Fisher Scientific), Foster City, CA, USA) and analyzed by 2^−ΔCt^. A list of primer sequences appears in Table S3.

### 4.11. ELISA

The Mouse CXCL10 ELISA Kit and Mouse CCL2 ELISA Kit from Peprotech (Cranbury, NJ, USA) were used for ELISA. Optical density (OD) values for each well were measured at 450 nm using a microplate reader.

### 4.12. Western Blot

Extracted total proteins from BMDMs, BV2 cells, and isolated mononuclear cells were run by electrophoresis with SDS-PAGE, then immunoblotting on nitrocellulose was performed. Blots detected with antibodies included the following: rabbit anti-ENO1 mAb (Abcam, Cambridge, UK), mouse anti-Tubulin antibody (Abmart, Inc., Shanghai, China), and anti-rabbit or anti-mouse IgG (H + L) secondary antibody (Abcam, Cambridge, UK). For antibodies stripping, blots were incubated with Restore PLUS Western Blot Stripping Buffer (Thermo Fisher Scientific, Waltham, MA, USA). The detection of protein bands was performed with an enhanced chemiluminescence kit from Thermo Fisher Scientific (Waltham, MA, USA), and ImageJ version 2.1.0 (NIH, Bethesda, MD, USA) was used to measure their density.

### 4.13. Drug Affinity Responsive Target Stability (DARTS) Assay

For discovering the drug target of PF, DARTS assay was performed according to the protocol by Lomenick [31]. In brief, BV2 cells in M1 polarization condition were lysed and protein concentrations were determined using the BCA Protein Assay Kit (Thermo Fisher Scientific, Waltham, MA, USA). Cell lysates were treated with either DMSO or PF (100 μM), incubated for 1 h, and digested with Pronase (Roche Diagnostics GmbH, Mannheim, Germany). The ratio of Pronase to protein were 1:100, 1:300, 1:1000, 1:2000, and 0 by mass. After mixing, cell lysate was incubated for 30 min at room temperature and stopped with protease inhibitors. The samples were loaded onto SDS-PAGE gels, stained with a Silver Stain kit (Thermo Fisher Scientific, Waltham, MA, USA), and the protein bands were cut out for LC-MS analysis by proteomics laboratory of Shanghai Jiao Tong University School of Medicine.

### 4.14. Microscale Thermophoresis (MST) Assay

MST experiments were conducted on a Monolith NT.115 system (NanoTemper Technologies GmbH, Munich, Germany) using standard treated capillary tubes. ENO1 was labeled using a Monolith MO-L018 kit (NanoTemper Technologies GmbH, Munich, Germany). Unlabeled PF was serially diluted from a concentration of 2.5 mM in the presence of 50 nM labeled ENO1 and loaded into premium capillaries. The data were analyzed using the MO Affinity Analysis software version 2.3 (NanoTemper Technologies GmbH, Munich, Germany).

### 4.15. Surface Plasmon Resonance (SPR) Assay

Binding affinities were measured using a Biacore^TM^ 8K instrument (Cytiva, Marlborough, MA, USA). His-tagged ENO1 (Cloud-Clone Corp, Wuhan, China) was immobilized on an NTA chip. PF, at concentrations from 100 μM to 3.125 μM, was injected at 40 μL/min for 120 s. During dissociation, the running buffer was injected at 30 μL/min for 120 s. Data analysis was conducted with Biacore™ Insight Software version 2.0.3 (Cytiva, Marlborough, MA, USA).

### 4.16. Molecular Docking of PF to ENO1

The ENO1 protein structure from the PDB and the PF compound file from TCMSP were imported into PyMOL version 2.5.0 (Schrödinger, LLC, New York, NY, USA), with irrelevant ligands and water molecules removed. AutoDock 4.2.6 software was used for docking simulation [32]. The protein was regularized to identify key amino acids in the predicted binding pocket. The optimal pose was chosen to analyze the interactions, and the pdbqt file was imported into PyMOL 2.5.0 to examine amino acid docking, binding energy, and bond distance. Finally, the ENO1 protein was displayed as a cartoon model.

### 4.17. Enolase Activity Assay

Primary or cultured cells (1 × 10^6^) were lysed with Enolase Assay Buffer (Sigma-Aldrich, St. Louis, MO, USA). Enolase activity of each sample and the standard curve were measured following the Enolase Activity Assay Kit (Sigma-Aldrich, St. Louis, MO, USA) instructions, and enzyme activity was calculated as per the manufacturer’s guidelines [33].

### 4.18. Seahorse Extracellular Flux Analysis

The glycolytic activity of BV2 cells and CD11b^+^ cells, isolated from CNS mononuclear cells, was assessed utilizing the Seahorse XF Glycolysis Stress Test Kit (Seahorse Biosciences (Agilent Technologies), North Billerica, MA, USA). The cells were cultured in microplates pre-coated with Poly-L-lysine at a density of 8 × 10^4^ cells/well and were allowed to adhere for at least 1 h. The assays were conducted by sequentially adding glucose, oligomycin, as well as 2-DG using an XF96 extracellular flux analyzer (Agilent, North Billerica, MA, USA), with data recorded automatically. Data analysis was performed using the Seahorse Wave Controller Software 2.4.2 (Agilent, North Billerica, MA, USA).

### 4.19. Cell Transfection for Eno1 Silencing and Overexpression

The siRNAs for *Eno1* were synthesized by Genomeditech (Shanghai, China):

si-NC was used as a negative control, and the sequence of si-NC is as follows: 5′-GGCTCTAGAAAAGCCTATGC-3′ (sense), and 5′-GGACUCUCGGAUUGUAAGAUU-3′ (anti-sense); the sequence of si-*Eno1* is as follows: 5′-GCAUUGGAGCAGAGGUUUATT-3′ (sense), and 5′-UAAACCUCUGCUCCAAUGCTT-3′ (anti-sense). Lipofectamine RNAiMAX Reagent (Thermo Fisher Scientific, Waltham, MA, USA) was used to transfect siRNAs into RAW 264.7 cells, and the cells were then incubated for 48 h. To generate *Eno1* overexpression plasmid, cDNA of mouse *Eno1* was cloned into plasmid pLVX-IRES-ZsGreen1. RAW 264.7 cells were transfected with *Eno1* overexpression plasmid or control plasmid by Lipofectamine 3000 (Thermo Fisher Scientific, Waltham, MA, USA) and incubated for 48 h. The levels of ENO1 expression were determined by western blot.

### 4.20. Statistical Analysis

SPSS Statistics version 26 and GraphPad Prism9 software were used for statistical analysis. One-way ANOVA followed by Dunnett’s multiple comparisons test were used to compare multiple groups. If the data complied with normal distribution, a two-tailed unpaired *t*-test was utilized. Otherwise, the Mann–Whitney U test was applied. Data were shown as the mean ± SEM. Statistical significance was defined as * *p* < 0.05, ** *p* < 0.01, *** *p* < 0.001; ns indicates no significance.

## 5. Conclusions

In summary, this study demonstrated for the first time that PF targets ENO1 to exert protective effects in EAE by alleviating microglia/macrophage activation, resisting inflammatory environment of CNS. PF directly binds to ENO1, leading to decreased ENO1 expression and enzyme activity with impaired glucose metabolism, thereby inhibiting M1 polarization of microglia/macrophages. PF is expected to be a potential drug candidate for MS, and ENO1 may be a novel therapeutic drug target.

## Data Availability

The data presented in this study are available in the article.

## Supplementary Materials

The following supporting information can be downloaded at: https://www.mdpi.com/article/10.3390/ijms26083677/s1.

## Abbreviations

This manuscript utilizes the following abbreviations:

BMDMs	bone marrow-derived macrophages
CFA	complete Freund’s adjuvant
CNS	central nervous system
DARTS	drug affinity responsive target stability
DMSO	dimethyl sulfoxide
DMTs	disease-modulatory therapies
EAE	experimental autoimmune encephalomyelitis
ECAR	extracellular acidification rate
ENO1	α-enolase
H&E	hematoxylin and eosin
HK2	hexokinase 2
LFB	luxol fast blue
LPS	lipopolysaccharides
MS	multiple sclerosis
MST	microscale thermophoresis
PF	paeoniflorin
PKM2	pyruvate kinase M2
SPR	surface plasmon resonance
WB	western blot
