# Supplementary material for: Paeoniflorin Directly Targets ENO1 to Inhibit M1 Polarization of Microglia/Macrophages and Ameliorates EAE Disease
Source: Int J Mol Sci. 2025 Apr 13;26(8):3677. doi: 10.3390/ijms26083677 (PMC12027182; doi:10.3390/ijms26083677)
Supplement: Supplementary file 1 [file ijms-26-03677-s001.zip › ijms-3556681-supplementary.pdf]

Supplymentary data

Table S1. Mass spectrometry of proteins from the DARTS samples

Protein Name	Score	PF sample #Spec PF	Ctrl sample #Spec CON	Spec ratio （PF/CON)
sp丨P17182丨ENOA_MOUSE	406.08	103	76	1.355263158
sp丨P52480丨KPYM_MOUSE	314.27	107	75	1.426666667
sp丨P40142丨TKT_MOUSE	286.04	58	52	1.115384615
sp丨P20029丨BIP_MOUSE	288.34	82	66	1.242424242
sp丨P63017丨HSP7C_MOUSE	297.49	80	61	1.31147541
sp丨Q9Z0N1丨IF2G_MOUSE	288.23	29	25	1.16
sp丨P09411丨PGK1_MOUSE	316.3	211	175	1.205714286

Table S2. The source and catalog number of the antibodies used in assays are provided

Antibodies	Source	Catalog number
CD45 Antibody-APC	Thermo Fisher Scientific	17-0451-82
CD3 Antibody-AF700		56-0032-82
CD4 Antibody-FITC		11-0041-85
CD4 Antibody-PerCP-Cyanine5.5		45-0042-82
CD11b Antibody-PE		12-0112-82
CD11b Antibody-APC		17-0112-82
IFN gamma Antibody-PE-Cyanine7		25-7319-82
IL-17A Antibody-PerCP-Cyanine5.5		45-7177-82
FOXP3 Antibody-APC		17-5773-82
CD206 Antibody-PE-Cyanine7		25-2061-82
Ki-67 Antibody-FITC		11-5698-82
CD86 Antibody-PE	BD Biosciences	561963
CD86 Antibody-BV421		564198

Table S3. Primer sequences used in qPCR

Name	5′-3′	Sequence
β-actin	Forward	TGTCCACCTTCCAGCAGATGT
	Reverse	AGCTCAGTAACAGTCCGCCTAG
Il-6	Forward	TACCACTTCACAAGTCGGAGGC
	Reverse	CTGCAAGTGCATCATCGTTGTTC
Il-1β	Forward	TCGCAGCAGCACATCAACAAGAG
	Reverse	AGGTCCACGGGAAAGACACAGG
Arg-1	Forward	CATTGGCTTGCGAGACGTAGAC
	Reverse	GCTGAAGGTCTCTTCCATCACC
Tgf-β	Forward	GGAGAAGTGAAGGATTACGAGC
	Reverse	CACACGATCTGGATGCCC
Ccl5	Forward	CCTGCTGCTTTGCCTACCTCTC
	Reverse	ACACACTTGGCGGTTCCTTCGA
Cxcl10	Forward	CCAAGTGCTGCGTCATTTTC
	Reverse	GGCTCGCAGGGATGATTTCAA
Ccl17	Forward	CGAGAGTGCTGCCTGGATTACT
	Reverse	GGTCTGCACAGATGAGCTTGCC
Ccl22	Forward	GTGGAAGACAGTATCTGCTGCC
	Reverse	AGGCTTGCGGCAGGATTTTGAG
Eno1	Forward	TGTGGCTGCCTCCGAGTTCTAC
	Reverse	ACTGGGTAGTTCTGGACGAAGGAC

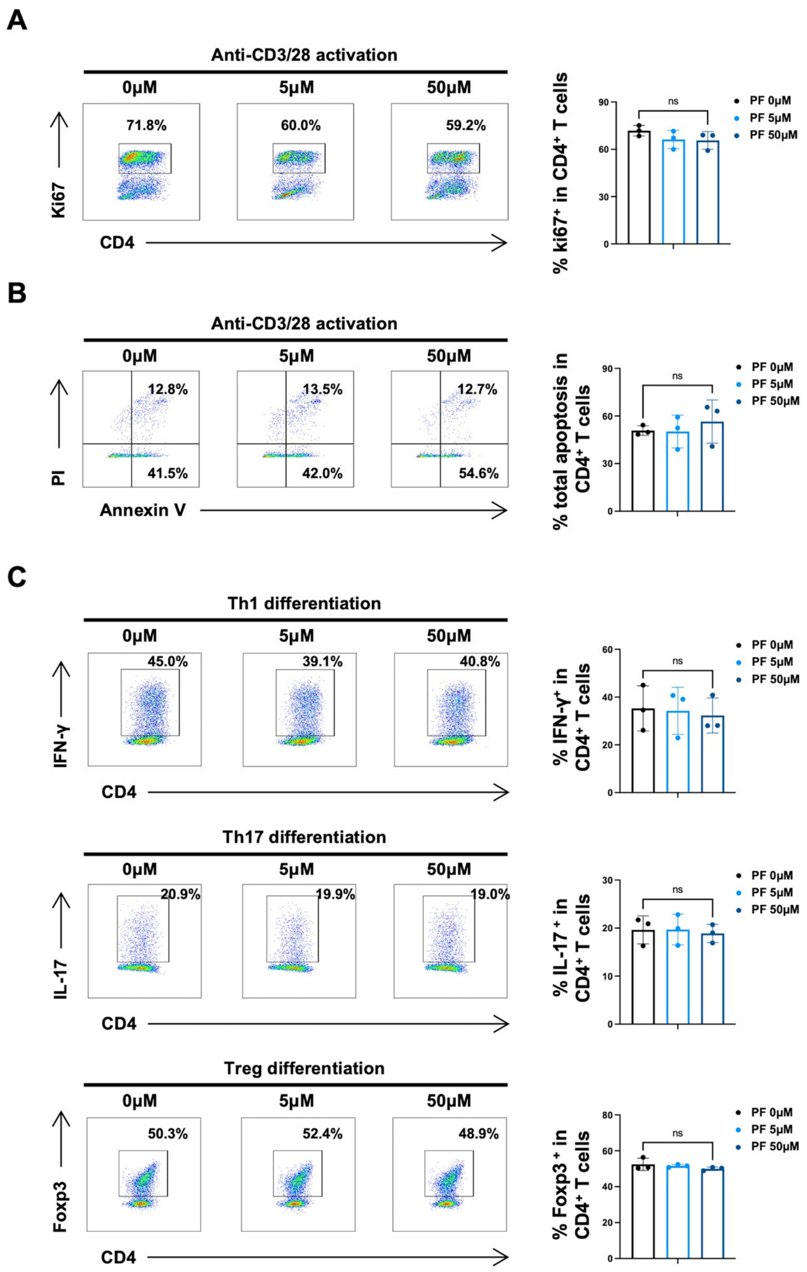

Figure S1. PF showed no obvious effect on $CD4^+$ T cell proliferation, apoptosis and differentiation. (A,B) $CD4^+$ T cells isolated from wild-type C57BL/6 mice were activated by anti-CD3e/CD28 mAbs for 48h with or without PF treatment. $Ki67^+$ percentage (A) and Annexin V^+ percentage (B) in $CD4^+$ T cells were analyzed by flow cytometry (n = 3). (C) $CD4^+$ T cells isolated from wild-type C57BL/6 mice were incubated under Th1, Th17, and Treg cell differentiation medium for 72h with or without PF treatment. The percentages of Th1 cells, Th17 cells and Treg cells in $CD4^+$ T cells were analyzed by flow cytometry (n = 3). Data are presented as the mean ± SEM. ns, no significance.

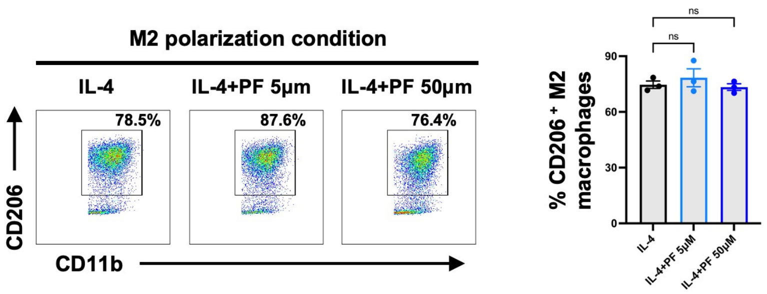

Figure S2. PF showed no obvious effect on M2 polarization of bone-marrow derived macrophages. BMDMs were treated with PF in M2 polarization condition (IL-4 20 ng/mL) for 24h. The percentages of CD206$^+$ M2 BMDMs were analyzed by flow cytometry (n = 3). Data are presented as the mean ± SEM. ns, no significance.
